# Exploring aortic morphology and determining variable-distance insertion lengths for fluoroscopy-free resuscitative endovascular balloon occlusion of the aorta (REBOA)

**DOI:** 10.1186/s13017-024-00557-4

**Published:** 2024-08-31

**Authors:** Jan C. van de Voort, Barbara B. Verbeek, Boudewijn L.S. Borger van der Burg, Rigo Hoencamp

**Affiliations:** 1 Department of Surgery, Alrijne Hospital, Simon Smitweg 1, Leiderdorp, 2353 GA The Netherlands; 2 Trauma Research Unit, Department of Surgery, Erasmus MC, University Medical Center Rotterdam, Rotterdam, The Netherlands; 3 Defense Healthcare Organization, Ministry of Defense, Utrecht, The Netherlands; 4 Department of Surgery, Leiden University Medical Center, Leiden, The Netherlands

**Keywords:** REBOA, Fluoroscopy-free, Pre-hospital, Aortic morphology, Aortic lengths

## Abstract

**Background (Rationale/Purpose/Objective):**

Resuscitative endovascular balloon occlusion of the aorta (REBOA) is used to temporary control non-compressible truncal hemorrhage (NCTH) as bridge to definitive surgical treatment. The dependence on radiography for safe balloon positioning is one factor that limits the extended use of REBOA in civilian and military pre-hospital settings. We aimed to determine standardized sex and age-based variable-distance catheter insertion lengths for accurate REBOA placement without initial fluoroscopic confirmation.

**Methods:**

Contrast enhanced CT-scans from a representative sample of a Dutch non-trauma population were retrospectively analyzed. Intravascular distances were measured from the bilateral common femoral artery access points (FAAP) to the middle of the aortic occlusion zones and accompanying boundaries. Means and 95% confidence intervals for the distances from the FAAPs to the boundaries and mid-zone III were calculated for all (combined) sex and age-based subgroups. Optimal insertion lengths and potentially safe regions were determined for these groups. Bootstrap analysis was performed in combination with a 40-mm long balloon introduction simulation to determine error-rates and REBOA placement accuracy for the general population.

**Results:**

In total, 1354 non-trauma patients (694 females) were included. Vascular distances increased with age and were longer in males. The iliofemoral trajectory was 7 mm longer on the right side. The optimal zone I catheter insertion length would be 430 mm. Optimal zone III catheter insertion lengths showed up to 30 mm difference, ranging between 234 and 264 mm. Statistically significant and potentially clinically relevant differences were observed between the anatomical distances and necessary introduction depths for each subgroup.

**Conclusion:**

This is the first study to compare aortic morphology and intravascular distances between combined sex and age-based subgroups. As zone III length was consistent, length variability and elongation seem to mainly originate in the iliofemoral trajectory and zone II. The optimal zone I catheter insertion length would be 430 mm. Optimal zone III catheter insertion ranged between 234 and 264 mm. These standardized variable-distance insertion lengths could facilitate safer fluoroscopy-free REBOA in austere, pre-hospital settings.

**Supplementary Information:**

The online version contains supplementary material available at 10.1186/s13017-024-00557-4.

## Introduction

Resuscitative endovascular balloon occlusion of the aorta (REBOA) is an endovascular technique for temporary control of non-compressible truncal hemorrhage (NCTH) to prevent hemodynamic collapse and lethal exsanguination before definitive surgical therapy. The REBOA-concept is straightforward; the aorta is occluded by inflating a balloon at the tip of an intraluminal catheter proximally of the suspected bleeding site. It increases coronary and cerebral perfusion pressures proximally and limits blood flow distally from the aortic occlusion balloon (AOB). REBOA can be performed with a ‘one-step-insertion’ method after obtaining common femoral artery (CFA) access and placement of an introducer sheath.

For the use of REBOA, the aorta is divided in three anatomical zones. Occlusion in zone I, from the left subclavian artery (LSA) to the celiac trunk (CT), is used for abdominal hemorrhage control or as pressure support in cardiopulmonary resuscitation for cardiac arrest of various etiology. Zone III, the infrarenal aorta, is targeted in case of pelvic or junctional inguinofemoral (groin) bleeding. Zone II comprises the visceral artery branches and is currently considered a no-go zone that should be actively avoided.

In general, catheter advancement and balloon positioning are guided by fluoroscopy to prevent the risk of balloon inflation in the aortic arch, zone II or the iliac arteries. Incorrect placement could lead to suboptimal bleeding control or cause iatrogenic cerebral, visceral or lower extremity ischemia. However, imaging is not always readily available at bed-side or in pre-hospital civilian and military combat settings. The dependence on radiographic imaging for safe balloon positioning limits the more widespread use of REBOA in these austere situations where it could eminently serve as a life-saving bridge to definitive hemorrhage control.

Different alternatives for balloon navigation have been investigated. These include substitute imaging techniques as portable ultrasonography [1–5] and alternative modalities as radiofrequency and magnetic tracking methods [6, 7]. Besides reports about successful ‘blind’ insertion [8–11], multiple methods have been proposed to enable imaging- and tracking-free REBOA. These include the use of external anatomical landmarks [12–17], linear prediction models based on torso length (TL) [18, 19], standardized fixed-distance catheter lengths based on large sample means [17, 20, 21] and mathematical multivariable prediction formulas [22, 23]. The mid-sternum landmark is a dependable bony reference point for zone I placement with 100% correlation rate and acceptable safety-margins [12, 13, 17]. In contrast, using the umbilicus for zone III would cause erroneous (partial) iliac artery balloon placement in 67–98% [14, 17, 18].

The use of an external landmark or fixed-distance insertion length seem useful for zone I considering its long length, but not suitable for zone III. No optimal zone III introduction depths have been determined yet. The available studies are based on different populations and propose inconclusive, divergent insertion lengths. Therefore, the need for a simple, fast and reliable insertion method to liberate REBOA from radiographic imaging remains.

The primary aim was to determine standardized variable-distance catheter insertion lengths based on the combination of sex and age. These variables are easy to determine and estimate on-scene. The secondary aim was to evaluate sex and age-related differences in aorto-iliofemoral morphology in a representative sample of the civilian population. Establishing broadly applicable variable-distance insertion lengths could contribute to safe REBOA use in austere settings.

## Method

### Study population

To obtain a representative convenience sample of the general civilian population, all non-trauma patients receiving a contrast-enhanced computed tomography scan of the abdomen, pelvic region and groins at the emergency department of a peripheral teaching hospital between Augustus 2019 and December 2022 were eligible for inclusion. For definitive in- or exclusion, all scans were assessed on contrast-enhancement quality and presence of image slices of the abdominal aorta from above the celiac trunk towards both femoral bifurcations. Patients with a history of (F)EVAR or prosthetic aortic graft were excluded.

### Central luminal line measurement method

**Fig. 1:**
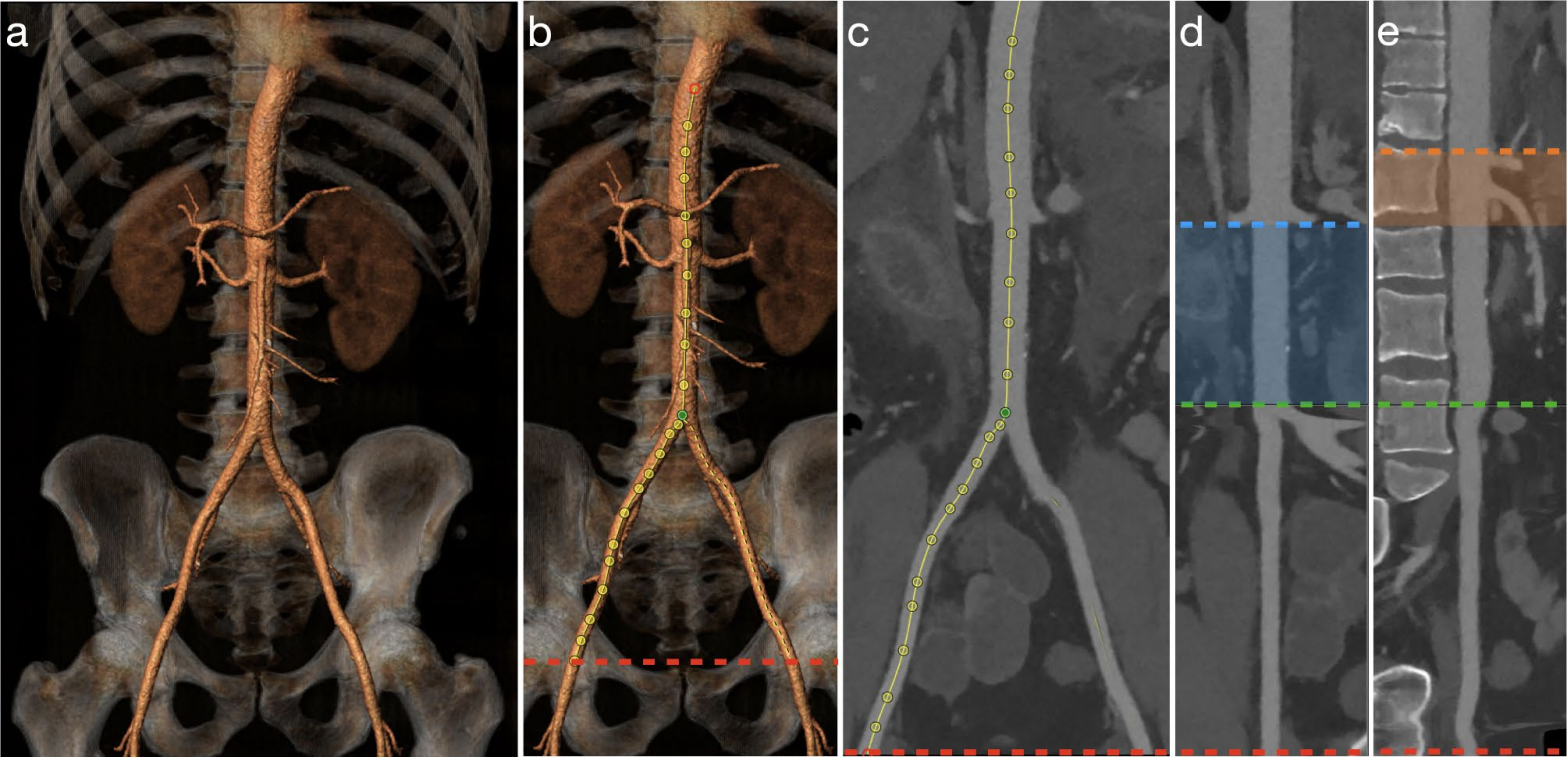
Representative images of the intravascular distances measurement method with a 3D-reconstruction of the aorta and iliofemoral trajectory (**a**), construction of a central luminal line (**b** & **c**) and the stretched vessel configuration in coronal (**d**) and sagittal (**e**) anatomical plane with zone III (blue shaded region) and II (orange shaded region). The dotted lines represent the mid-femoral head (red), aortic bifurcation (green), distal origin of lowest renal artery (blue) and proximal origin of celiac trunk (orange) levels

The software program 3Mensio Vascular™ (Pie Medical Imaging BV, Maastricht, The Netherlands) was used to create a 3D-reconstruction of the aorta and iliofemoral trajectories and construct a central luminal line (CLL) within this vasculature (Fig. 1). By measuring along the central axis of the aorto-iliofemoral lumen, zone lengths and intravascular distances from the femoral arterial access points (FAAP) to the zone boundaries could be determined. The right and left CFA at the mid-femoral head level were chosen as the FAAP and starting points for the distance measurements [17–19, 23]. The REBOA-zones were defined by the major aortic branches which represent the zone boundaries. Zone III length was the distance between the aortic bifurcation (AB, inferior boundary of zone III) and the distal origin of the lowest main renal artery (RA, superior boundary of zone III). Zone II extended from the lower RA to the CT. Zone I ranged from the proximal origin of the CT (inferior boundary of zone I) to the distal origin of the LSA (superior boundary of zone I). Aortic diameters and the depth of the left CFA, defined as the shortest distance from the skin, were also measured. Two authors (JvdV and BV) performed the measurements.

### Calculating fixed-distance catheter insertion lengths

For all subgroups, mean CLL-distances from the bilateral FAAP to all zone boundaries and mid-zone III with accompanying 95% (2 SD) confidence interval limits were calculated. Hereby the optimal mean catheter insertion lengths for zone III balloon positioning and potentially safe regions could be determined for all subgroups. Our analyzed scans did not include the thoracic aorta. To calculate the optimal zone I insertion length for our sample, the shortest absolute zone I length from all previously published studies was used to minimize the risk of LSA occlusion.

Subsequently, we simulated the introduction of the 40-mm long ER-REBOA™ (PryTime Medical™ Devices, Inc., Boerne, Texas) to determine malposition percentages. The calculated catheter insertion lengths corresponded with the middle of the balloon with 20-mm balloon extension in both cranial and caudal direction. Previously proposed insertion lengths were applied to our sample to compare out-of-zone placement rates [17, 20, 21, 23, 24].

### Statistical analysis

Deidentified patient characteristics, total body height and -weight and distance measurements were registered in a Microsoft Excel^®^ spreadsheet, version 16.66.1 (Microsoft, Redmond, Washington, USA). (Combined) sex-and age-based subgroups were created. Four age-groups were arbitrarily chosen; 18–30, 30–50, 50–70 and 70–90 years. Statistical analyses were performed using Statistical Package for the Social Sciences (SPSS^®^), version 29 (IBM corporation, Armonk, New York, USA) and GraphPad Prism^®^, version 9.5.0 (GraphPad Software Inc., San Diego, California, USA). Inter- and intra-observer reliability and consistency testing was performed at the start and end of the data collection for 30 patients each time using the intraclass correlation efficient and Cronbach’s alpha. Missing data were computed based on available age, body height and -weight data of other patients.

Data normality testing was done with histograms and Shapiro–Wilk tests. Continuous (quantitative) data were presented using mean ± standard deviation (SD) or median with interquartile range (IQR). Differences in measured distances between the two sexes regardless of age were compared with an unpaired Student’s t-test or Mann-Whitney U test. Comparison between (combined sex and) age-based subgroups was done with the one-way ANOVA with Bonferroni correction for multiple testing or the Kruskall-Wallis test. For each subgroup, bootstrap analysis with 10,000 sample simulations was performed to determine the proportion of patients for whom balloon placement at the calculated mean insertion length would have resulted in a ‘too high’ or ‘too low’ out-of-zone placement [25, 26]. For analyses, a p value < 0.05 was considered significant.

## Results

### Population demographics

In total, 1354 non-trauma patients (694 females) were included after excluding 64 individuals because of various reasons (Table 1 & supplemental Fig. 1). Missing body height and weight data were computed for 264 patients (19.5%). Inter-observer reliability testing asserted there was no significant difference between the two measurers.

### General sex- and age-based length variability

Up to the diaphragm, sex and age-based length variability and aortic elongation mainly occurs in zone II and the iliofemoral trajectory. Regardless of sex or insertion side, comparing the youngest with the oldest age group, the age-related mean distance increase from the FAAPs to the aortic bifurcation was 12 mm (male 15, female 9), to the lowest RA 13 mm (male 18, female 9) and to the CT 21 mm (male 26, female 18). Zone III showed a consistent length of ± 89 mm (male 91, female 87) regardless of age. Zone II, with a length of 37–49 mm, showed an age-related increase of 8 mm in both sexes, which translates to a 20–25% increase over the years (Tables 2 and 3 & supplemental Table 1). On average, the right iliofemoral vasculature was 7 mm longer compared with the left side regardless of sex or age.

**Table 1:** Study population patient characteristics, sex-based CLL distance measurements from the femoral arterial access points and aortic bifurcation to mid-zone III and zone boundaries, aortic diameters & CFA depth in mm

	Total (*n* = 1354) mean (SD) median [IQR]		Male (*n* = 660) mean (SD)	Female (*n* = 694) mean (SD)	*p* value
Age (years)	59.6 (17.7)	61.8 [26]	59.1 (18.0)	60.1 (17.4)	< 0.001
Total body height (cm)	172.2 (9.3)	172.0 [11]	177.7 (7.6)	167.0 (7.5)	< 0.001
Body weight (kg)	80.5 (17.3)	80.0 [18]	85.8 (17.2)	75.6 (15.8)	< 0.001
BMI (kg/m^2^)	27.1 (5.4)	26.8 [5.6]	27.1 (4.7)	27.1 (5.9)	0.88
CFA right - AoBif	208 (21.7)	207 [27]	214 (22.9)	203 (19.1)	< 0.001
CFA left - AoBif	201 (20.9)	199 [26]	206 (22.3)	196 (18.1)	< 0.001
CFA right - mid zone III	253 (22.2)	251 [27]	259 (23.2)	247 (19.2)	< 0.001
CFA left - mid zone III	246 (21.2)	244 [27]	252 (22.5)	240 (18.1)	< 0.001
CFA right – renal artery	296 (24.2)	295 [30]	305 (25.1)	290 (21.0)	< 0.001
CFA left – renal artery	290 (23.2)	288 [29]	297 (24.3)	283 (19.8)	< 0.001
CFA right – celiac trunk	343 (26.3)	340 [34]	352 (27.6)	334 (21.9)	< 0.001
CFA left – celiac trunk	335 (25.4)	333 [32]	344 (26.9)	327 (20.8)	< 0.001
AoBif – mid zone III	44 (6.1)	44 [8]	45 (6.3)	43 (5.7)	< 0.001
AoBif – RA (zone III length)	89 (12.2)	89 [17]	91 (12.6)	87 (11.4)	< 0.001
AoBif – celiac trunk	134 (14.6)	134 [19]	138 (15.2)	131 (13.2)	< 0.001
RA – CT (zone II length)	46 (8.6)	45 [11]	47 (9.1)	44 (7.8)	< 0.001
Diameter mid zone III	18.8 (3.9)	18.3 [4.0]	19.9 (3.7)	17.6 (3.7)	< 0.001
Diameter mid zone II	21.6 (3.2)	21.5 [4.0]	22.7 (3.2)	20.5 (2.8)	< 0.001
Skin to CFA left	34.7 (17.1)	31.5 [19.0]	31.8 (13.9)	37.4 (19.2)	< 0.001

**Table 2:** Age-based CLL distance measurements from the femoral arterial access points to mid-zone III and zone boundaries, aortic diameters & CFA depth in mm for males

Male (mm)	18–30 yrs (*n* = 53) mean (SD)	30–50 yrs (*n* = 136) mean (SD)	50–70 yrs (*n* = 259) mean (SD)	70–90 yrs (*n* = 212) mean (SD)
CFA right - AoBif	202 (17.2)	210 (16.8)	215 (23.9)	217 (24.8)
CFA left - AoBif	194 (17.7)	202 (16.7)	208 (24.6)	210 (22.1)
CFA right - mid zone III	247 (16.9)	255 (16.6)	260 (23.8)	264 (25.8)
CFA left – mid zone III	239 (17.3)	247 (16.7)	253 (24.1)	256 (23)
CFA right – renal artery	292 (18.2)	300 (18.3)	304 (25.1)	311 (28.4)
CFA left – renal artery	285 (18.4)	292 (18.6)	298 (25.2)	303 (25.8)
CFA right – celiac trunk	333 (19.9)	337 (20.1)	353 (27.4)	359 (30.4)
CFA left – celiac trunk	326 (20.4)	330 (20.3)	346 (27.5)	352 (27.9)
Zone III length (AoBif- RA)	91 (10.6)	90 (11.6)	89 (12.3)	93 (13.5)
Zone II length (RA– CT)	41 (8.9)	45 (6.9)	49 (9.7)	49 (8.6)
Diameter mid zone III	15.8 (1.9)	18.6 (1.9)	19.7 (2.4)	22.2 (4.7)
Diameter mid zone II	18.0 (2.2)	21.3 (2.1)	22.6 (2.4)	24.8 (3.2)
Skin to CFA	25.2 (12.6)	31.8 (13.6)	31.6 (14.5)	33.6 (13.3)

**Table 3:** Age-based CLL distance measurements from the femoral arterial access points to mid-zone III and zone boundaries, aortic diameters & CFA depth in mm for females

Female (mm)	18–30 yrs (*n* = 52) mean (SD)	30–50 yrs (*n* = 140) mean (SD)	50–70 yrs (*n* = 264) mean (SD)	70–90 yrs (*n* = 238) mean (SD)
CFA right - AoBif	198 (15.1)	203 (15.9)	201 (18.1)	207 (21.7)
CFA left - AoBif	190 (14.6)	196 (15.7)	195 (17.6)	199 (19.9)
CFA right - mid zone III	242 (15.9)	246 (15.9)	244 (18.0)	251 (22.1)
CFA left – mid zone III	234 (15.8)	239 (16.0)	238 (17.2)	243 (19.9)
CFA right – renal artery	285 (18.0)	289 (17.9)	287 (19.6)	294 (23.9)
CFA left – renal artery	277 (18.2)	282 (18.1)	282 (18.7)	287 (21.6)
CFA right – celiac trunk	322 (19.1)	332 (18.2)	333 (20.4)	340 (24.4)
CFA left – celiac trunk	314 (19.4)	324 (18.4)	327 (19.5)	332 (22.3)
Zone III length (AoBif- RA)	87 (9.7)	86 (11.5)	87 (11.4)	87 (11.6)
Zone II length (RA - CT)	37 (5.2)	43 (6.9)	45 (8.3)	46 (7.3)
Diameter mid zone III	14.6 (2.0)	16.5 (1.8)	17.4 (2.8)	19.2 (4.8)
Diameter mid zone II	16.7 (2.0)	19.4 (2.1)	20.3 (2.0)	22.2 (2.8)
Skin to CFA	28 (12.1)	38.6 (24.2)	39.2 (17.9)	36.9 (17.7)

### Sex-based insertion lengths for zone III

After correction for multiple testing, all measured distances within the specific age-groups differed significantly between the sexes. For males, regardless of age, the optimal zone III catheter insertion length as calculated from the right and left CFA was 259 mm (95% CI 257.3-260.8) and 252 mm (95% CI 250.1-253.5), respectively. For females, regardless of age, the optimal insertion length from the right CFA was 247 mm (95% CI 245.0-247.9) and 240 mm (95% CI 238.2-240.9) from the left CFA (Table 1).

### Age-based insertion lengths for zone III

Mean mid-zone III distances from the bilateral FAAPs showed significant differences between the four arbitrarily chosen age groups regardless of sex, except between the 30-50- and 50-70-years age groups. As measured from the right side, the optimal catheter insertion length for the age group of 18–30 years was 244 mm, for 30–70 years 252 mm and for 70–90 years 257 mm. From the left side, the optimal catheter insertion lengths were 237, 243–246 and 249 mm, respectively (supplemental Table 1).

### Combined sex- and age-based insertion lengths for zone III

Significant differences were observed when comparing between sexes, age groups and insertion sides. Longer distances were consistently observed in males, elderly and on the right side (Tables 2, 3 and 4). For males, a 17 mm age-related increase of mean mid-zone III distance was observed between the youngest and oldest age group from both insertion sides. On the right side, optimal insertion lengths were 247, 255, 260 and 264 mm, respectively. On the left side these were 239, 243, 253 and 256 mm, respectively.

**Fig. 2:**
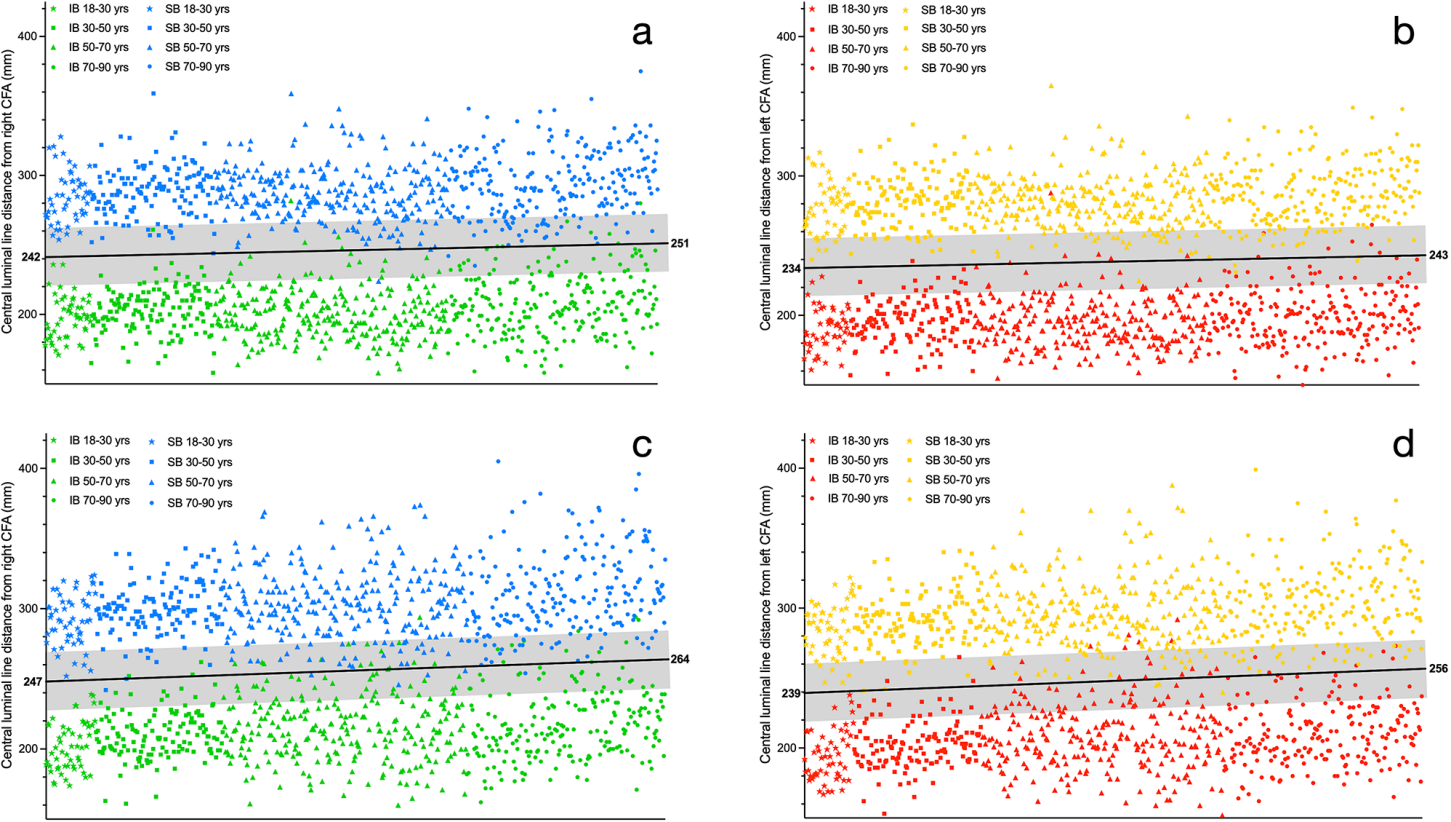
Simulation of a 4-cm long balloon placement with 20 mm extension in cranial and caudal direction (transparent grey region) based on combined sex- and age-based variable-distance catheter insertion lengths (black line) for insertion from the right (**a** & **c**) and left (**b** & **d**) FAAPs. Individual superior (blue & yellow symbols) and inferior (green & orange symbols) zone III boundaries for females (**a** & **b**) and males (**c** & **d**) are plotted

For females, a bilateral 9 mm increase of mean mid-zone III distance was observed over time. On the right side the distances were 242, 246, 244 and 251 mm while on the left side 234, 239, 238 and 243 mm, respectively. Sex-based elongation differences became larger with increased age. A maximum of 30 mm difference in mean zone III distance exists between the subgroups (Table 4; Fig. 2). For both sexes, not all age-based insertion lengths differed significantly from each other (supplemental Table 2).

**Table 4:** Optimal mean mid-zone III catheter insertion lengths of the combined sex and age-based subgroups from left and right FAAPs in mm

AFC TO MID-ZONE III (mm, 95% CI)		18–30 yrs	30–50 yrs	50–70 yrs	70–90 yrs
CFA left	Female	234 (229.2–238.0)	239 (236.1-241.5)	238 (236.0-240.2)	243 (240.4-245.5)
	Both sexes	237 (233.3-239.8)	243 (241.0-245.0)	246 (243.5-247.3)	249 (247.0-251.1)
	Male	239 (234.6-244.3)	247 (244.4-250.1)	253 (250.0-255.9)	256 (252.7–259.0)
CFA right	Female	242 (237.0-245.9)	246 (243.3-248.7)	244 (241.8-246.1)	251 (247.6-253.2)
	Both sexes	244 (241.0-247.5)	251 (248.5-252.5)	252 (249.7-253.6)	257 (254.4–259.0)
	Male	247 (242.3-251.7)	255 (252.3–258.0)	260 (256.7-262.5)	264 (260.2-267.1)

### Standardized fixed-distance insertion length for zone I

The minimum and maximum distance from the FAAPs to the inferior boundary of zone I were 280 and 460 mm, respectively. The shortest measured zone I length from all previously published studies was 190 mm. Hypothetically, the minimal absolute distance from the FAAPs to the LSA would therefore be 470 mm. For our sample, the optimal catheter insertion length would be 430 mm (20 mm cranial and caudal extension). This would cause a ‘too low’ error with partial zone II occlusion in maximal 1.8% of patients with at least a 2 cm proximal error margin to the LSA and 0% LSA occlusion.

### Depth of common femoral artery

The mean distance from skin to the left CFA was 34.7 (SD 17.1) mm. In all age-groups, higher values were observed in females. Depth of the femoral artery was directly proportional to BMI (*r* = 0.654, R^2^ = 0.428, *p* < 0.001). 591 patients (43.6%) were in the overweight range (BMI 25–30) and 294 patients (21.7%) had obesity (BMI > 30).

### Comparison with previous studies

High out-of-zone error-rates up to 89% were observed when applying previously proposed fixed-distance zone III catheter insertion lengths to our different subgroups. Our variable-distance insertion lengths would lead to combined out-of-zone error-rates between 13 and 33%. Sub analysis showed that sex- or age-based only lengths caused higher error-rates in the youngest age-groups, but similar malposition rates in older age-groups compared with combined sex- and age-based lengths (supplemental file).

## Discussion

The optimal zone I catheter insertion length would be 430 mm. Optimal zone III insertion lengths showed up to 30 mm difference, ranging between 234 and 264 mm. Currently, the JTSCPG recommends introduction depths from the CFA of 460 mm for zone I and 280 mm for zone III, regardless of insertion side or patient characteristics as age, sex and TL. Although a one-size-fits-all strategy may be the most practical, a high percentage of patients would receive incorrect balloon positioning when using a solitary fixed-distance insertion length for zone III. This could result in inadequate hemorrhage control or iatrogenic limb and organ ischemia.

For those at risk of hemodynamic collapse, immediate action is required to achieve blood pressure stabilization and prevent exsanguination. In austere settings, REBOA could be the only viable option to maintain sufficient cardiac and cerebral perfusion. Bringing REBOA closer to the point-of-injury could prove lifesaving as endovascular alternative to external aortic clamping through thoracotomy [27]. In our recently published Delphi study, the panel recommended the initiation of a randomized clinical trial investigating REBOA use in civilian pre-hospital settings [28]. To robustly implement REBOA in (remote) pre-hospital trauma care, abolishing the need for additional imaging equipment and requiring minimal patient information is necessary.

### Previously proposed standardized lengths

Fixed-distance catheter insertion lengths seem usable for zone I, but nor for zone III balloon positioning. Through analysis of 280 CT-scans of an equally mixed-sex civilian cohort (mean age 39 years), Pezy et al. defined a common segment in zone III between 236 and 256 mm as measured from the upper border of the pubic symphysis with a general predicted prevalence of 95% [20]. In 59 REBOA-patients Meyer et al. observed a median zone III insertion length of 29 (range 21–36) cm. Pezy’s predicted safe regions would have been located at least partially out of their measured zone boundaries in 50%.

Borger et al. included 250 patients (151 males, mean age 69 years) to compare age- and sex-based differences in central vasculature [17]. Measuring from the mid-section of the right CFA, they found mean mid-zone III distances of 274 (SD 28.0) mm for males and 251 (SD 23.3) mm for females. Divided in age-groups, mean mid-zone III distances were 251 (20–50 years), 263 (51–70 years) and 264 (71–94 years) mm regardless of sex. Applying the JTSCPG zone III introduction depth, accurate positioning occurred in only 38.0% with mainly ‘too low’ errors. Olsen et al. found similar lengths in a study with 100 patients (62 males, mean age 78 years). Distance from the left CFA (3 cm below mid-inguinal point) to mid-zone III was 27 (SD 2.0) cm for males and 25 (SD 1.8) cm for females [21]. An optimal 95% safe region would be located between 26 and 27 cm, but would still have led to six out-of-zone placements. In 177 males (median age 23 years, Afghan nationals and deployed US military personnel) Morrison et al. measured median mid-zone III distances from the right and left CFA at the mid-femoral head level of 232 (IQR 21) and 228 (IQR 22) mm, respectively [19]. Ethnicity had no effect on prediction accuracy.

Eliason et al. evaluated 1525 CT-scans of male and female trauma patients (1114 males, median age 31 years) [23]. The proposed fixed-distance insertion length for zone III was 28 cm when inserted from both FAAPs at the mid-femoral head level. Despite using a relatively simple equation to adjust for individual TL, the 33.3% out-of-zone placement rate could only be reduced to 26.3%. For zone III, variable-distance insertion lengths ranged from 22.5 cm to 32.5 cm, increasing 0.5 cm for 1 cm increase in TL. A 35% out-of-zone error-rate for zone III positioning was seen when simulating the then applicable JTSCPG depth of 27 cm.

These studies did not evaluate nor compare combined sex and age-based subgroups, propose divergent lengths and show accuracy discrepancies. Applying the generally longer introduction depths than those found in the current study would lead to high out-of-zone error-rates with extraordinary outliers for certain subgroups in our sample. Although not all of our subgroup mid-zone III lengths differed significantly, sex- or age-based only introduction depths would lead to higher erroneous zone II error-rates with possible RA or CT occlusion in younger patients. A more patient-tailored introduction method facilitates improved REBOA placement accuracy.

### Zone III length, CFA depth & iliofemoral length variability

We found a rather consistent mean zone III length of approximately 89 mm. Previously reported lengths were between 83 and 104 mm with no mentioned significant sex differences. Only Borger et al. previously differentiated between age-groups and reported a significant difference between the two youngest and the oldest age-groups; 88, 96 and 104 mm, respectively. We observed a mean CFA depth of 34.7 mm, although with high individual variability. Similarly, Stannard and Eliason et al. observed median depths of 35 (29–41) mm and 37 (27–43) mm, while Pezy et al. reported a mean depth of 24 (10) mm. In accordance with previous studies, reporting differences between 4 and 9 mm, we found a significantly longer iliofemoral trajectory on the right side with an overall length difference of 7 mm regardless of sex or age.

### Practical usability & feasibility

When possible, correct balloon position should always be confirmed. However, it has not been clarified yet whether (partial) zone II occlusion causes significant long-term detrimental side-effects compared to zone I or even zone III occlusion as is commonly believed [29, 30]. Since erroneous zone II occlusion would provide better bleeding-control than iliac artery occlusion, ‘too low’ errors should at least be avoided to prevent suboptimal bleeding-control combined with the needless risk of iatrogenic limb ischemia or possible dissection due to overinflation. Partial occlusion, either by partial and intermittent inflation or using innovative balloons, could potentially mitigate ischemia-related complications [31–36]. Partial zone II occlusion, most frequently limited to RA occlusion, seems practically unavoidable for a small percentage of the population, even when using variable zone III insertion lengths. Future studies should evaluate if and how erroneous zone II positioning is tolerated by investigating the possible local and systemic adverse effects of CT, superior mesenteric artery and RA occlusion.

In contrast to most studies, we took the balloon length in consideration for calculation and comparison of out-of-zone error-rates. These rates were much higher when including the balloon length. Extraordinarily high error-rates were seen when mutually comparing and applying the previously proposed insertion lengths and landing zones that do not take into account the age and sex-based variability. We observed up to almost a balloon length difference in mean mid-zone III distances between our subgroups. Therefore, we advocate that introduction depths should indeed be adjusted for sex and age as firstly proposed by Borger et al. to ensure more precise positioning in the youngest patients [17]. By applying different colored segments on conventional ABO-catheters, corresponding with specific subgroups, our variable-distance catheter insertion model could be simply implemented in daily practice.

### Limitations

There are limitations to this study. Firstly, we aimed to prevent selection bias by including all patients receiving a CT-scan within a pre-determined time period regardless of age, sex and scan-indication. However, due to the location of the hospital in a semi-rural area we have not been able to include a wide variety of race or ethnicity. Secondly, the accuracy of determining the FAAP precisely at the mid-femoral head level cannot be matched in real-life. While we anticipate that this will not cause problems for zone I considering its long length, it could cause extra out-of-zone errors when targeting zone III. Thirdly, a stretched-vessel-configuration does not fully reflect the catheter pathway in tortuous vasculature. Lastly, the depth of the CFA varied greatly within the sample. In daily practice this will also be affected by the needle insertion angle and puncture site location. To achieve accurate balloon positioning, this depth must be taken into consideration and added to the insertion length. Ultrasonography could be used for both tissue thickness determination as well as FAAP localization.

By investigating a large, heterogenous group, we have gained better understanding of aorto-iliofemoral morphology. A more patient-tailored insertion-method seems feasible, but could also lead to a more complex model. A balance must be found between the number of subgroups and acceptable error-rates. Insertion models irrespective of body height or torso length are more straightforward. Our reproducible strategy only requires on-scene sex-determination and age-estimation without the need for time-consuming measurements or arduous calculations. Future studies are required to validate the proposed catheter insertion lengths and determine the degree of possible partial zone II occlusion.

## Conclusion

We characterized the morphology of the abdominal aorta and identified optimal REBOA catheter insertion lengths for zone I and III balloon occlusion in a sample of the general civilian population. This is the first combined sex and age-based comparison of aortic intravascular distances. Significantly longer distances were seen in males, elderly and on the right side. Optimal zone III insertion lengths ranged between 234 and 264 mm. As the length of zone III is very consistent, length variability and elongation below the diaphragm seems to originate in the iliofemoral trajectory and zone II. The universal zone I insertion length is 430 mm. Standardized variable-distance catheter insertion lengths based on sex and age could facilitate safer fluoroscopy-free REBOA in pre-hospital and resource limited settings.

### Electronic supplementary material

Below is the link to the electronic supplementary material.

Supplementary Material 1

Supplementary Material 2

## Data Availability

No datasets were generated or analysed during the current study.

## Declarations

### Disclaimer

The opinions or assertions contained herein are the private views of the authors and are not to be construed as official or reflecting the views of the Dutch Ministry of Defense or any other agency of the Dutch government. Several authors are employees of the Dutch government.

## Abbreviations

REBOA: Resuscitative endovascular balloon occlusion of the aorta
NCTH: Non-compressible truncal hemorrhage
AOB: Aortic occlusion balloon
CFA: Common femoral artery
LSA: Left subclavian artery
CT: Celiac trunk
TL: Torso length
(F)EVAR: (Fenestrated) endovascular aneurysm repair
CLL: Central luminal line
FAAP: Femoral arterial access point
AB: Aortic bifurcation
RA: Renal artery
SD: Standard deviation
IQR: Interquartile range
BMI: Body mass index
JTSCPG: Joint Trauma System Clinical Practice Guideline
ABO: Aortic balloon occlusion
