# Supplementary material for: Exploring aortic morphology and determining variable-distance insertion lengths for fluoroscopy-free resuscitative endovascular balloon occlusion of the aorta (REBOA)
Source: World J Emerg Surg. 2024 Aug 31;19:29. doi: 10.1186/s13017-024-00557-4 (PMC11365199; doi:10.1186/s13017-024-00557-4)
Supplement: Supplementary file 1 — Supplementary Material 1 [file 13017_2024_557_MOESM1_ESM.pdf]

1. SUPPLEMENTAL MATERIAL

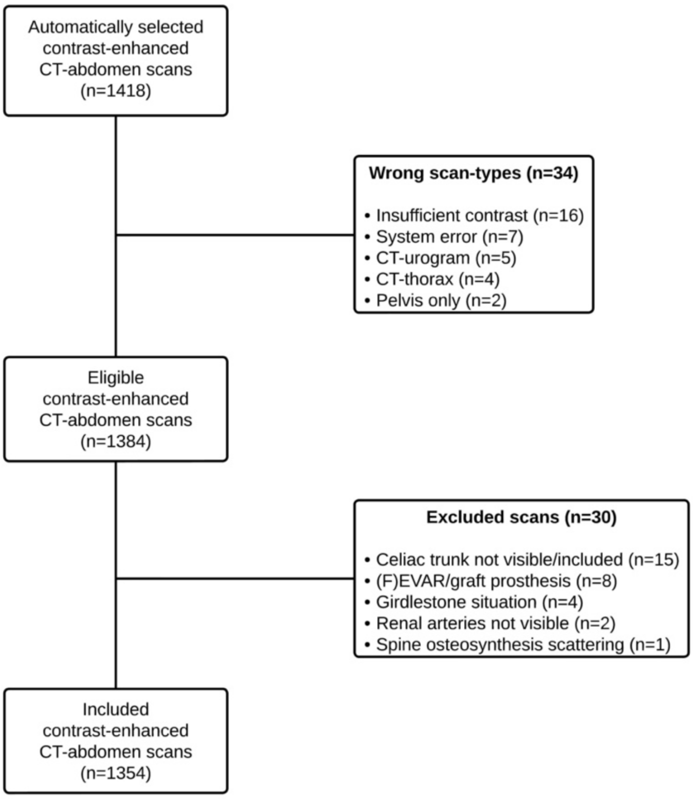

Supplemental figure 1 Study inclusion and exclusion flowchart of automatically selected contrast-enhanced CT-abdomen scans

AGE SUBGROUPS REGARDLESS OF SEX	18-30 yrs (n = 105) *mean (SD)*	30-50 yrs (n = 276) *mean (SD)*	50-70 yrs (n = 523) *mean (SD)*	70-90 yrs (n = 450) *mean (SD)*
CFA right - AoBif	200 (16.3)	207 (16.7)	208 (22.4)	212 (23.8)
CFA left - AoBif	192 (16.4)	199 (16.6)	202 (22.4)	204 (21.6)
CFA right - mid zone 3	244 (16.6)	251 (16.9)	252 (22.4)	257 (24.8)
CFA left – mid zone 3	237 (16.8)	243 (16.9)	246 (22.2)	249 (22.5)
CFA right – renal artery	289 (18.5)	294 (18.9)	296 (24.0)	302 (27.4)
CFA left – renal artery	281 (18.7)	287 (19.1)	289 (23.5)	294 (25.0)
CFA right – celiac trunk	328 (20.3)	338 (20.3)	343 (26.1)	349 (29.0)
CFA left – celiac trunk	320 (20.7)	330 (20.3)	336 (25.7)	341 (26.8)
Zone 3 length (AoBif- RA)	89 (10.4)	88 (11.7)	88 (11.9)	90 (12.9)
Zone 2 length (RA - CT)	39 (7.5)	44 (7.0)	47 (9.2)	47 (8.0)
Diameter mid zone III	15.2 (2.0)	17.6 (2.1)	18.5 (2.9)	20.6 (5.0)
Diameter mid zone II	17.4 (2.2)	20.4 (2.3)	21.4 (2.5)	23.4 (3.3)
Skin to CFA	26.6 (12.5)	35.2 (20.0)	35.4 (16.7)	35.4 (15.9)

Supplemental table 1 Age-based CLL distance measurements from the arterial access points to mid-zone III and zone boundaries, aortic diameters & CFA depth regardless of sex

			1-2 *p-value*	1-3 *p-value*	1-4 *p-value*	2-3 *p-value*	2-4 *p-value*	3-4 *p-value*
**Both sexes**	**AFC left**	**mid-zone III**	.01	.001	<.001	1.0	.008	.01
		**celiac trunk**	<.001	<.001	<.001	.04	<.001	.02
	**AFC right**	**mid-zone III**	.02	.01	<.001	1.0	.05	.005
		**celiac trunk**	<.001	<.001	<.001	.37	<.001	.006
**Male**	**AFC left**	**mid-zone III**	.11	<.001	<.001	.26	.004	.52
		**celiac trunk**	.05	<.001	<.001	.005	<.001	.35
	**AFC right**	**mid-zone III**	.07	.001	<.001	.94	.049	.78
		**celiac trunk**	.03	<.001	<.001	.03	<.001	.48
**Female**	**AFC left**	**mid-zone III**	.29	.66	.007	1.0	.58	.03
		**celiac trunk**	.02	<.001	<.001	1.0	.005	.02
	**AFC right**	**mid zone III**	.39	1.0	.02	.79	.89	.003
		**celiac trunk**	.02	.009	<.001	1.0	.01	.003

Supplemental table 2 Overview of statistical comparison of the calculated distances from the bilateral arterial access points to mid-zone III and celiac trunk for the different combined sex- and age-based subgroups and insertion sides with age groups 1 (18-30 yrs), 2 (30-50 yrs), 3 (50-70 yrs) and 4 (70-90 yrs)

**SEX SUBGROUPS REGARDLESS OF AGE**	**Total** (n = 1354)		**Male** (n = 660)		**Female** (n = 694)	
	Minimum	Maximum	Minimum	Maximum	Minimum	Maximum
CFA right - AoBif	158	294	160	294	158	282
CFA left - AoBif	150	292	152	292	150	288
CFA right - mid zone 3	191	344	202	344	191	344
CFA left – mid zone 3	192	333	196	333	192	327
CFA right – renal artery	224	407	242	405	224	375
CFA left – renal artery	225	399	240	399	225	365
CFA right – celiac trunk	282	460	282	454	287	460
CFA left – celiac trunk	280	448	281	448	280	405
Zone 3 length (AoBif- RA)	57	142	57	142	58	127
Zone 2 length (RA - CT)	21	74	23	74	21	70

Supplemental table 3 Minimum and maximum values of CLL distances from the FAAPs to mid-zone & zone boundaries and CFA depth in mm regardless of age

**AGE SUBGROUPS REGARDLESS OF SEX**	**18-30 years** (n = 105)		**30-50 years** (n = 276)		**50-70 years** (n = 523)		**70-90 years** (n = 450)	
	Min	Max	Min	Max	Min	Max	Min	Max
CFA right - AoBif	171	239	158	262	158	294	158	294
CFA left - AoBif	161	238	153	265	152	292	150	273
CFA right - mid zone 3	212	281	201	310	191	334	197	344
CFA left – mid zone 3	200	280	197	303	192	333	193	332
CFA right – renal artery	252	328	242	359	224	374	235	407
CFA left – renal artery	240	322	241	341	225	388	231	399
CFA right – celiac trunk	295	372	282	400	287	435	287	460
CFA left – celiac trunk	280	362	281	397	284	447	282	448
Zone 3 length (AoBif- RA)	68	121	58	128	57	124	57	142
Zone 2 length (RA - CT)	24	69	21	66	23	74	23	70

Supplemental table 4 Age-based minimum and maximum values of CLL distances from the FAAPs to mid-zone & zone boundaries and CFA depth in mm regardless of sex

MALE	18-30 yrs (n = 53)		30-50 yrs (n = 136)		50-70 yrs (n = 259)		70-90 yrs (n = 212)	
	Min	Max	Min	Max	Min	Max	Min	Max
CFA right - AoBif	174	239	161	262	160	294	162	294
CFA left - AoBif	167	238	153	265	152	292	162	273
CFA right - mid zone 3	215	281	202	300	203	334	211	344
CFA left – mid zone 3	208	280	197	303	196	333	208	332
CFA right – renal artery	252	324	242	343	246	374	254	405
CFA left – renal artery	247	322	241	341	240	388	255	399
CFA right – celiac trunk	296	372	282	400	287	435	295	454
CFA left – celiac trunk	285	362	281	397	289	447	298	448
Zone 3 length (AoBif- RA)	74	121	61	128	57	122	57	142
Zone 2 length (RA - CT)	24	69	31	66	23	74	23	69

Supplemental table 5 Age-based minimum and maximum values of CLL distances from the FAAPs to mid-zone & zone boundaries and CFA depth in mm for males

FEMALE	18-30 yrs (n = 52)		30-50 yrs (n=140)		50-70 yrs (n = 264)		70-90 yrs (n = 238)	
	Min	Max	Min	Max	Min	Max	Min	Max
CFA right - AoBif	171	236	158	261	158	282	158	280
CFA left - AoBif	161	228	157	239	155	288	150	265
CFA right - mid zone 3	213	279	201	310	191	321	197	344
CFA left – mid zone 3	200	268	200	288	192	327	193	304
CFA right – renal artery	254	328	244	359	224	359	235	407
CFA left – renal artery	240	317	244	337	225	365	231	349
CFA right – celiac trunk	295	366	293	396	287	399	287	460
CFA left – celiac trunk	280	355	284	374	284	405	282	401
Zone 3 length (AoBif- RA)	68	108	58	122	58	124	63	127
Zone 2 length (RA - CT)	26	50	21	60	26	66	30	70

Supplemental table 6 Age-based minimum and maximum values of CLL distances from the FAAPs to mid-zone & zone boundaries and CFA depth in mm for females
