# Supplementary material for: Exploring aortic morphology and determining variable-distance insertion lengths for fluoroscopy-free resuscitative endovascular balloon occlusion of the aorta (REBOA)
Source: World J Emerg Surg. 2024 Aug 31;19:29. doi: 10.1186/s13017-024-00557-4 (PMC11365199; doi:10.1186/s13017-024-00557-4)
Supplement: Supplementary file 2 — Supplementary Material 2 [file 13017_2024_557_MOESM2_ESM.pdf]

Out-of-zone error rates: bootstrap analysis and comparison with previous studies

Bootstrapping analysis with simulation of a 40-mm long balloon placement in zone III showed that our variable-distance insertion lengths would lead to combined out-of-zone error-rates between 13-33% for the specific subgroups. Slightly higher percentages for males, but an almost equal distribution between the two error-types were observed (tables 1,2, 3 & 4).

Simulating a 40-mm long balloon placement in zone III, high out-of-zone error-rates were observed when applying the previously proposed fixed-distance catheter insertion lengths to the different (combined) sex and age subgroups of our sample (tables 5,6 & 7). The sex-based lengths of Olsen and Borger et al. showed mainly ‘too high’ error-rates up to 64% for specific subgroups, in particular for younger males when inserted from the left side. For females, ‘too high’ error-rates ranged between 16-39%. In contrast, the Joint Trauma System Clinical Practice Guideline (JTSCPG) depths caused ‘too high’ error-rates of 41-89%, with highest rates in younger females. Overall, ‘too low’ error-rates ranged between 0-15% for the different introduction depths. When not taking into account the balloon length, Olsen and Borger et al. ‘too high’ error-rates were drastically lowered to the 0-28% range, in particular for women. For the JTSCPG lengths, ‘too high’ error-rates dropped to 10-42% for male and 27-52% for female subgroups. Our overall error-rates would have dropped to 0-4% for 18-50 years and to 2-8% for 50-90 years with maximum errors for women of 1.4 % and 4.6%, respectively (tables 1,2, 3 & 4).

Sub analysis showed that insertion lengths only based on sex or age caused higher error-rates in the youngest age groups, but similar malposition rates in older age groups as compared with combined sex- and age-based lengths (data and tables available on request).

Applying previously proposed zone I catheter insertion lengths to our sample, the balloon would have been (partially) positioned in zone II in 0.2% to 3.8% of patients regardless of sex or age (table 8).

SEX SUBGROUPS REGARDLESS OF AGE		Mean mid-zone 3 (mm, 95% CI)	'Too low' error (%, with consideration of balloon length)	'Too high' error (%, with consideration of balloon length)	'Too low' error (%, mid-zone III point)	'Too high' error (%, mid-zone III point)
**All** (n = 1354)	CFA RIGHT	253 (251.4-253.8)	12.9	14.9	3.4	1.3
	CFA LEFT	246 (244.4-246.7)	12.3	13.7	3.0	1.0
**Male** (n = 660)	CFA RIGHT	259 (257.3-260.8)	13.0	14.3	3.8	1.5
	CFA LEFT	252 (250.1-253.5)	12.6	13.2	3.8	1.2
**Female** (n = 694)	CFA RIGHT	247 (245.0-248.0)	11.0	12.7	2.2	0.6
	CFA LEFT	240 (238.2-241.0)	10.0	11.1	2.0	0.6

Supplemental file, table 1 Out-of-zone error rate percentages for females, males and whole sample. Rates are presented for simple mid-zone III distances and placement simulation with inclusion of the balloon length (40 mm). A 'too low' error would lead to (partial) iliac artery occlusion, a 'too high' error to (partial) zone II occlusion.

**AGE SUBGROUPS REGARDLESS OF SEX**		**Mean mid-zone 3 (mm, 95% CI)**	**‘Too low’ error (%, with consideration of balloon length)**	**‘Too high’ error (%, with consideration of balloon length)**	**‘Too low’ error (%, mid-zone III point)**	**‘Too high’ error (%, mid-zone III point)**
**18-30 yrs** (n = 105)	CFA RIGHT	244 (241.0-247.5)	6.6	11.3	0.0	0.0
	CFA LEFT	237 (233.3-239.8)	7.5	11.3	0.9	0.0
**30-50 yrs** (n = 276)	CFA RIGHT	251 (248.5-252.5)	6.9	10.6	1.5	1.1
	CFA LEFT	243 (241.0-245.0)	8.4	12.7	1.1	0.7
**50-70 yrs** (n = 523)	CFA RIGHT	252 (249.7-253.6)	14.3	14.6	4.3	1.6
	CFA LEFT	246 (243.5-247.3)	13.2	13.4	3.7	0.8
**70-90 yrs** (n = 450)	CFA RIGHT	257 (254.4-259.0)	14.9	17.9	3.7	2.2
	CFA LEFT	249 (247.0-251.1)	12.5	16.9	3.5	1.3

Supplemental file, table 2 Out-of-zone error rate percentages for age subgroups, regardless of sex. Rates are presented for simple mid-zone III distances and placement simulation with inclusion of the balloon length (40 mm). A ‘too low’ error would lead to (partial) iliac artery occlusion, a ‘too high’ error to (partial) zone II occlusion.

MALE (n=660)		Mean mid-zone 3 (mm, 95% CI)	'Too low' error (%, with consideration of balloon length)	'Too high' error (%, with consideration of balloon length)	Total out-of-zone error rate (%)	'Too low' error (%, mid-zone III point)	'Too high' error (%, mid-zone III point)
**18-30 yrs** (n = 53)	CFA RIGHT	247 (242.3-251.7)	9.4	9.4	18.8	0.0	0.0
	CFA LEFT	239 (234.6-244.3)	7.5	9.4	16.9	0.0	0.0
**30-50 yrs** (n = 136)	CFA RIGHT	255 (252.3-258.0)	7.3	8.8	16.1	0.7	1.5
	CFA LEFT	247 (244.4-250.1)	8.1	7.4	15.5	2.2	1.5
**50-70 yrs** (n = 259)	CFA RIGHT	260 (256.7-262.5)	15.3	15.7	31	5.1	1.6
	CFA LEFT	253 (250.0-255.9)	13.4	14.6	28	5.5	2.4
**70-90 yrs** (n = 212)	CFA RIGHT	264 (260.2-267.1)	14.2	16.6	30.9	4.2	2.3
	CFA LEFT	256 (252.7-259.0)	12.9	15.2	28.1	2.8	0.9

Supplemental file, table 3 Age based out-of-zone error rate percentages for males. Rates are presented for simple mid-zone III distances and placement simulation with inclusion of the balloon length (40 mm). A 'too low' error would lead to (partial) iliac artery occlusion, a 'too high' error to (partial) zone II occlusion.

**FEMALE (n=694)**		**Mean mid-zone 3 (mm, 95% CI)**	**'Too low' error (%, with consideration of balloon length)**	**'Too high' error (%, with consideration of balloon length)**	**Total out-of-zone error rate (%)**	**'Too low' error (%, mid-zone III point)**	**'Too high' error (%, mid-zone III point)**
**18-30 yrs** (n = 52)	CFA RIGHT	242 (237.0-245.9)	5.6	11.3	17	0.0	0.0
	CFA LEFT	234 (229.2-238.0)	5.7	7.6	13.3	0.0	0.0
**30-50 yrs** (n = 140)	CFA RIGHT	246 (243.3-248.7)	5.7	12.2	18	0.7	0.7
	CFA LEFT	239 (236.1-241.5)	7.2	12.9	20.2	0.7	0.0
**50-70 yrs** (n = 264)	CFA RIGHT	244 (241.8-246.1)	10.7	10.7	21.4	1.9	0.4
	CFA LEFT	238 (236.0-240.2)	8.4	8.0	16.4	2.3	0.4
**70-90 yrs** (n = 238)	CFA RIGHT	251 (247.6-253.2)	14.6	18.3	32.9	2.1	1.2
	CFA LEFT	243 (240.4-245.5)	11.3	12.5	23.8	2.5	2.1

Supplemental file, table 4 Age based out-of-zone error rate percentages for females. Rates are presented for simple mid-zone III distances and placement simulation with inclusion of the balloon length (40 mm). A 'too low' error would lead to (partial) iliac artery occlusion, a 'too high' error to (partial) zone II occlusion.

OUT-OF-ZONE ERROR RATES SEX SUBGROUPS REGARDLESS OF AGE		'Too low' error (with consideration of balloon length)			'Too high' error (with consideration of balloon length)			'Too low' error (mid-zone III point)			'Too high' error (mid-zone III point)		
		Borger	*JTSCPG Eliason*	*Olsen*	*Borger*	*JTSCPG Eliason*	*Olsen*	*Borger*	*JTSCPG Eliason*	*Olsen*	*Borger*	*JTSCPG Eliason*	*Olsen*
**Male** (n = 660)	CFA RIGHT	30 (4.5%)	24 (3.6%)	44 (6.7%)	224 (33.9%)	302 (45.8%)	180 (27.3%)	9 (1.4%)	4 (0.6%)	11 (1.7%)	58 (8.8%)	94 (14.2%)	40 (6.1%)
	CFA LEFT	20 (3.0%)	15 (2.3%)	28 (4.2%)	311 (47.1%)	393 (59.6%)	260 (39.4%)	4 (0.6%)	2 (0.3%)	7 (1.1%)	101 (15.3%)	151 (22.9%)	71 (10.8%)
**Female** (n = 694)	CFA RIGHT	51 (7.3%)	4 (0.6%)	54 (7.8%)	124 (17.9%)	491 (70.8%)	113 (16.3%)	8 (1.2%)	1 (0.1%)	9 (1.3%)	9 (1.3%)	222 (32.0%)	8 (1.2%)
	CFA LEFT	21 (3.0%)	2 (0.3%)	26 (3.7%)	185 (26.7%)	568 (81.8%)	177 (25.5%)	4 (0.6%)	1 (0.1%)	5 (0.7%)	22 (3.2%)	305 (44.0%)	19 (2.7%)

Supplemental file, table 5 Out-of-zone error rate percentages of previously proposed catheter insertion lengths applied to our sample. Rates are presented for simple mid-zone III distances and placement simulation with inclusion of the balloon length (40 mm). A 'too low' error would lead to (partial) iliac artery occlusion, a 'too high' error to (partial) zone II occlusion.

**OUT-OF-ZONE ERROR RATES** **MALE (n=660)**		**'Too low' error (with consideration of balloon length)**			**'Too high' error (with consideration of balloon length)**			**'Too low' error (mid-zone III point)**			**'Too high' error (mid-zone III point)**		
		**Borger**	***JTSCPG Eliason***	***Olsen***	**Borger**	***JTSCPG Eliason***	***Olsen***	**Borger**	***JTSCPG Eliason***	***Olsen***	**Borger**	***JTSCPG Eliason***	***Olsen***
**18-30 yrs** (n = 53)	CFA RIGHT	0	0	0	29 (54.7%)	32 (60.4%)	22 (41.5%)	0	0	0	8 (15.1%)	13 (24.5%)	6 (11.3%)
	CFA LEFT	0	0	0	34 (64.2%)	41 (77.4%)	29 (54.7%)	0	0	0	15 (28.3%)	22 (41.5%)	12 (22.6%)
**30-50 yrs** (n = 136)	CFA RIGHT	1 (0.7%)	1 (0.7%)	3 (2.2%)	45 (33.1%)	70 (51.5%)	32 (23.5%)	0	0	0	12 (8.8%)	13 (9.6%)	6 (4.4%)
	CFA LEFT	1 (0.7%)	1 (0.7%)	1 (0.7%)	75 (55.1%)	97 (71.3%)	58 (42.6%)	0	0	0	17 (12.5%)	31 (22.8%)	13 (9.6%)
**50-70 yrs** (n = 259)	CFA RIGHT	15 (5.8%)	12 (4.6%)	20 (7.7%)	86 (33.2%)	113 (43.6%)	77 (29.7%)	4 (1.5%)	1 (0.4%)	4 (1.5%)	26 (10.0%)	41 (15.8%)	21 (8.1%)
	CFA LEFT	13 (5.0%)	9 (3.5%)	17 (6.6%)	123 (47.5%)	148 (57.1%)	105 (40.5%)	4 (1.5%)	2 (0.8%)	6 (2.3%)	38 (14.7%)	62 (23.9%)	28 (10.8%)
**70-90 yrs** (n = 212)	CFA RIGHT	14 (6.6%)	11 (5.2%)	21 (9.9%)	64 (30.2%)	87 (41.0%)	49 (23.1%)	5 (2.4%)	3 (1.4%)	7 (3.3%)	12 (5.7%)	27 (12.7%)	7 (3.3%)
	CFA LEFT	6 (2.8%)	5 (2.4%)	10 (4.7%)	79 (37.3%)	107 (50.5%)	68 (32.1%)	0	0	1 (0.5%)	31 (14.6%)	36 (17.0%)	18 (8.5%)

Supplemental file, table 6 Out-of-zone error rate percentages of previously proposed catheter insertion lengths applied divided per age-group in males. Rates are presented for simple mid-zone III distances and placement simulation with inclusion of the balloon length (40 mm). A 'too low' error would lead to (partial) iliac artery occlusion, a 'too high' error to (partial) zone II occlusion. Borger: 274 mm, Olsen: 270 mm, JTSCPG/Eliason: 280 mm

**OUT-OF-ZONE ERROR RATES**		**'Too low' error (with consideration of balloon length)**			**'Too high' error (with consideration of balloon length)**			**'Too low' error (mid-zone III point)**			**'Too high' error (mid-zone III point)**		
**FEMALE (n=694)**		**Borger**	***JTSCPG Eliason***	***Olsen***	**Borger**	***JTSCPG Eliason***	***Olsen***	**Borger**	***JTSCPG Eliason***	***Olsen***	**Borger**	***JTSCPG Eliason***	***Olsen***
**18-30 yrs** (n = 52)	CFA RIGHT	2 (3.8%)	0	2 (3.9%)	12 (23.1%)	42 (80.8%)	10 (19.2%)	0	0	0	0	22 (42.3%)	0
	CFA LEFT	0	0	0	20 (38.5%)	46 (88.5%)	19 (36.5%)	0	0	0	3 (5.8%)	27 (51.9%)	3 (5.8%)
**30-50 yrs** (n = 140)	CFA RIGHT	4 (2.9%)	1 (0.7%)	4 (2.9%)	22 (15.7%)	105 (75.0%)	20 (14.3%)	1 (0.7%)	0	1 (0.7%)	1 (0.7%)	38 (27.1%)	1 (0.7%)
	CFA LEFT	2 (1.4%)	0	2 (1.4%)	35 (25.0%)	120 (85.7%)	34 (24.3%)	0	0	0	4 (2.9%)	61 (43.6%)	4 (2.9%)
**50-70 yrs** (n = 264)	CFA RIGHT	12 (4.5%)	1 (0.4%)	13 (4.9%)	46 (17.4%)	202 (76.5%)	44 (16.7%)	3 (1.1%)	1 (0.4%)	3 (1.1%)	5 (1.9%)	90 (34.1%)	5 (1.9%)
	CFA LEFT	7 (2.7%)	1 (0.4%)	9 (3.4%)	70 (26.5%)	226 (85.6%)	66 (25.0%)	1 (0.4%)	1 (0.4%)	1 (0.4%)	8 (3.0%)	125 (47.4%)	7 (2.7%)
**70-90 yrs** (n = 238)	CFA RIGHT	33 (13.9%)	2 (0.8%)	35 (14.7%)	44 (18.5%)	142 (59.7%)	39 (16.4%)	4 (1.7%)	0	5 (2.1%)	3 (1.3%)	72 (30.3%)	2 (0.8%)
	CFA LEFT	12 (5.0%)	1 (0.4%)	15 (6.3%)	60 (25.2%)	176 (74.0%)	58 (24.4%)	3 (1.3%)	0	4 (1.7%)	7 (2.9%)	92 (38.7%)	5 (2.1%)

Supplemental file, table 7 Out-of-zone error rate percentages of previously proposed catheter insertion lengths applied divided per age-group in females. Rates are presented for simple mid-zone III distances and placement simulation with inclusion of the balloon length (40 mm). A 'too low' error would lead to (partial) iliac artery occlusion, a 'too high' error to (partial) zone II occlusion. Borger: 251 mm, Olsen: 250 mm, JTSCPG/Eliason: 280 mm

ZONE I<br>ALL PATIENTS	AFC right	AFC left
**JTSCPG** (460 mm)	5 (0.4%)	2 (0.2%)
**Eliason et al.** (480 mm)	0 (0.0%)	0 (0.0%)
**Olsen et al.** (430-480 mm)	25 (1.9%)	12 (0.9%)
**Pezy et al.*** (414-474 mm)	52 (3.8%)	33 (2.4%)

*Supplemental file, table 8 Out-of-zone error percentage for previously proposed zone I catheter insertion lengths applied to this study sample population, * measured from pubic symphysis*
